# Error in Affiliations

**DOI:** 10.1001/jamanetworkopen.2020.7853

**Published:** 2020-05-08

In the Invited Commentary, “Importance of Quality-of-Life Measurement Throughout the Disease Course,”^1^ published on March 4, 2020, Hilde M. Buiting, PhD, incorrectly indicated her current affiliation as “Antoni van Leeuwenhoek, Netherlands Cancer Institute.” The correct affiliation should have been indicated as “Amsterdam, the Netherlands.” This article has been corrected.

## References

[1] Buiting HM, Olthuis G Importance of quality-of-life measurement throughout the disease course. JAMA Netw Open. 2020;3(3):e200388. doi:10.1001/jamanetworkopen.2020.0388 32129862

